# Association of stent diameter and target vessel revascularization in patients undergoing percutaneous coronary intervention: a secondary retrospective analysis based on a Chinese cohort study

**DOI:** 10.1186/s12872-021-02212-1

**Published:** 2021-08-21

**Authors:** Tiancheng Xu, Beili Feng, Zaixing Zheng, Licheng Li, Weifang Zeng, Dongjuan Wang, Lin Zhang, Hengdong Li

**Affiliations:** 1 Department of Cardiology, Hwa Mei Hospital, University of Chinese Academy of Sciences, No. 41 Northwest Street, Haishu District, Ningbo, 315000 Zhejiang China; 2 Ningbo Institute of Life and Health Industry, University of Chinese Academy of Sciences, Ningbo, 315000 Zhejiang China

**Keywords:** Stent diameter, Target vessel revascularization, Percutaneous coronary intervention

## Abstract

**Background:**

In the treatment of coronary heart disease, target vessel revascularization (TVR) has attracted increasing attention as an efficient means of percutaneous coronary intervention (PCI). The purpose of this study was to explore the association between stent diameter and TVR in patients undergoing PCI.

**Methods:**

This was a secondary retrospective analysis involving patients with PCI with at least one stent implanted. Information was obtained from the Dryad Digital Repository. Multivariable logistic regression models, interaction analyses, subgroup analyses and piecewise linear regression models were used to evaluate the association between stent diameter and TVR.

**Results:**

A total of 2522 patients were eventually enrolled in this study, of which 122 (4.8%) had undergone TVR. Significant positive associations were observed between stent diameter and TVR (continuous: odds ratio [OR] 0.485, 95% confidence interval [CI] 0.305–0.773, P = 0.002; categorical variable: T2 vs. T1, OR 0.541, 95% CI 0.348–0.843; T3 vs. T1, OR 0.520, 95% CI 0.334–0.809; P for trend = 0.005). The association remained stable in the fully adjusted model (continuous: OR 0.526, 95% CI 0.306–0.902, P = 0.020; categorical variable: T2 vs. T1, OR 0.510, 95% CI 0.310–0.839; T3 vs. T1, OR 0.585, 95% CI 0.352–0.973; P for trend = 0.042). Among the subgroups of differing clinical presentations, stent diameter was a powerful protective factor for TVR, especially in the delayed PCI group (P for interaction = 0.002). The association was highly consistent across all the other subgroups studied (all P for interaction > 0.05). In the piecewise linear regression model, the need for TVR decreased with an increase in stent diameter when this ranged between 2.5 and 2.9 mm (OR 0.01, 95% CI: 0.01–0.13, P < 0.001).

**Conclusions:**

A large stent diameter is a powerful protective factor for TVR in PCI patients, especially in the delayed PCI group. This “bigger-is-better” protective effect is remarkable in stents with diameter 2.5–2.9 mm.

## Background

Since stents are widely used in the treatment of coronary heart disease, the efficacy of percutaneous coronary intervention (PCI), including target vessel revascularization (TVR), has attracted increasing attention. The rate of TVR has shown a downward trend with improvements in stents and techniques [1–4]. Previous studies have reported several effective predictors of TVR, such as age, diabetes mellitus, stent length and small vessel lesions [5–8]. However, data from registries on long-term follow-up are sparse concerning the relationship between stent diameter and TVR. The purpose of this study was to explore the association between stent diameter and TVR in patients undergoing PCI.

## Methods

### Data source

The datasets generated and analyzed during the current study are available from the Dryad Digital Repository, [https://datadryad.org/resource/doi:10.5061/dryad.13d31].

### Study design and participants

**Fig. 1:**
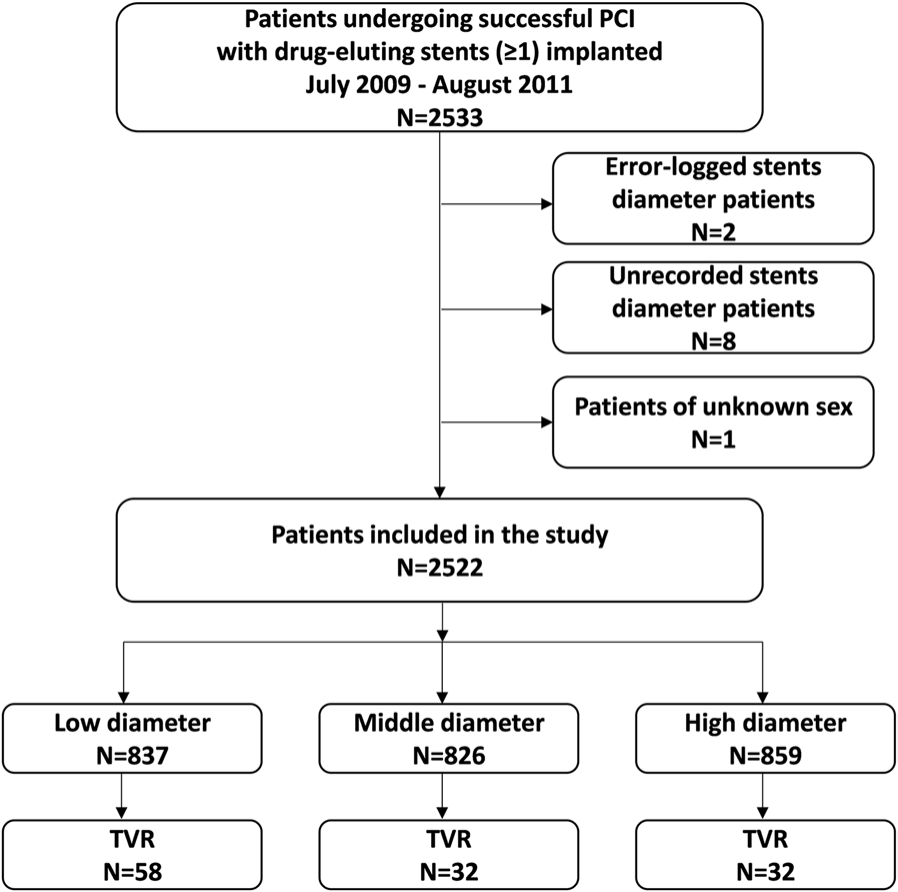
A flow chait of the inclusion and exclusion of patients

This was a secondary retrospective analysis based on a cohort study. Patients undergoing PCI with at least one stent implanted between July 2009 and August 2011 at a single high-volume PCI center in China were included in the study. Standard methods were used for acquiring coronary angiographic analyses and PCI. All patients were continuously enrolled unless: (1) stent diameter was unrecorded; (2) stent diameter was demonstrably wrong; (3) sex was unspecified. Any other detail of the cohort has been described in the original article [9]. Figure 1 shows the details of the inclusions and exclusions.

### Outcome and data collection

The primary clinical outcome of the study was TVR, which is defined as the need for repeat PCI or coronary artery bypass grafting (CABG) in the target vessel. Demographic data, medical history, laboratory data, angiographic and procedural information were extracted from the previously mentioned database.

### Statistical analyses

All participants were categorized separately into three tertiles according to stent diameter. Continuous variables were presented as mean ± SD or median [interquartile range] for variables with a skewed distribution. Categorical variables were presented as numbers (n) and percentages (%). The characteristics of the study population according to the diameter of the stent trisector were compared using a one-way analysis of variance (ANOVA) or Kruskal–Wallis test for continuous variables, and χ^2^ test for categorical variables.

Univariate analysis was performed to detect the possible risks associated with TVR. We carried out multiple logistic regression models to evaluate the association between stent diameter and TVR. Both non-adjusted and multivariate adjusted models were applied. We selected adjusted confounders on the basis of their associations with outcomes or a change in effect estimate of more than 10%. Interaction and subgroup analyses were performed for the different groups. All confounding variables were adjusted for each stratification, except the stratification factor itself. Furthermore, the threshold effect of stent diameter on TVR was explored using piecewise linear regression according to the smoothing plot.

All tests were two-sided and a P value less than 0.05 was considered significant. Analyses were performed using the statistical software packages R (http://www.R-project.org, The R Foundation) and Empower Stats (http://www.empowerstats.com, X&Y Solutions, Inc., Boston, MA).

## Results

### Baseline characteristics of study population

As shown in Fig. 1, among the 2533 patients who underwent successful PCI with at least one stent implanted, 11 patients were excluded based on the exclusion criteria. A total of 2522 patients were eventually enrolled in this study, of which 122 (4.8%) underwent TVR. Individuals were classified into three groups according to stent diameter: T1 (≤ 2.85 mm), T2 (2.86–3.2 mm) and T3 (≥ 3.21 mm). Table 1 depicts the baseline characteristics of the overall patients and by tertiles of the stent diameter. Overall, the mean age was 60.0 ± 11.1 years and 1715 (68.0%) of the patients were men. Participants with smaller stent diameters were more likely to be older, male predominant and smokers. They were also more likely to have hypertension, diabetes mellitus and myocardial infarction.

**Table 1:** Characteristics of study patients

Characteristics	Overall	Tertiles of stent diameter of 2522 patients			
		T1 (≤ 2.85)	T2 (2.86–3.20)	T3 (≥ 3.21)	P value
N	2522	837	826	859	
TVR	122 (4.8%)	58 (6.9%)	32 (3.9%)	32 (3.7%)	0.003
*Demographics*					
Age (years)	60.0 ± 11.1	61.3 ± 10.6	60.5 ± 10.8	58.2 ± 11.6	< 0.001
Male, n (%)	1715 (68.0%)	518 (61.9%)	566 (68.5%)	631 (73.5%)	< 0.001
*Medical history*					
Hypertension, n (%)	1240 (49.2%)	447 (53.4%)	415 (50.2%)	378 (44.1%)	< 0.001
Diabetes mellitus, n (%)	520 (20.6%)	217 (25.9%)	170 (20.6%)	133 (15.5%)	< 0.001
Atrial fibrillation, n (%)	50 (2.0%)	12 (1.4%)	17 (2.1%)	21 (2.4%)	0.322
Stroke, n (%)	133 (5.3%)	45 (5.4%)	54 (6.5%)	34 (4.0%)	0.06
III degree AVB, n (%)	8 (0.3%)	4 (0.5%)	1 (0.1%)	3 (0.3%)	0.506
COPD, n (%)	22 (0.9%)	3 (0.4%)	11 (1.3%)	8 (0.9%)	0.100
Heart failure, n (%)	294 (11.7%)	109 (13.0%)	98 (11.9%)	87 (10.1%)	0.173
Cardiac shock, n (%)	4 (0.2%)	0 (0.0%)	1 (0.1%)	3 (0.3%)	0.281
PVD, n (%)	6 (0.2%)	3 (0.4%)	2 (0.2%)	1 (0.1%)	0.542
MI, n (%)	233 (9.2%)	84 (10.0%)	89 (10.8%)	60 (7.0%)	0.017
Smoking, n (%)	811 (32.2%)	243 (29.0%)	259 (31.4%)	309 (36.0%)	0.008
Prior CABG, n (%)	21 (0.8%)	8 (1.0%)	6 (0.7%)	7 (0.8%)	0.874
Prior PCI, n (%)	169 (6.7%)	59 (7.0%)	58 (7.0%)	52 (6.1%)	0.65
*Medication*					
Aspirin, n (%)	2487 (98.7%)	823 (98.3%)	814 (98.7%)	850 (99.1%)	0.406
Clopidogrel, n (%)	2415 (95.9%)	803 (96.2%)	792 (95.9%)	820 (95.6%)	0.429
β-blocker, n (%)	1711 (67.8%)	552 (65.9%)	575 (69.6%)	584 (68.0%)	0.277
ACEI, n (%)	1350 (53.6%)	433 (51.7%)	453 (54.9%)	464 (54.0%)	0.407
CCB, n (%)	598 (23.7%)	211 (25.2%)	192 (23.2%)	195 (22.7%)	0.444
Statin, n (%)	2293 (90.9%)	750 (89.6%)	762 (92.3%)	781 (90.9%)	0.172
*Laboratory tests*					
LDL-C, mmol/L	2.7 ± 0.9	2.7 ± 0.9	2.7 ± 0.9	2.7 ± 0.9	0.638
HDL-C, mmol/L	1.1 ± 0.3	1.1 ± 0.3	1.1 ± 0.3	1.1 ± 0.3	0.549
TC, mmol/L	4.3 ± 1.1	4.3 ± 1.0	4.2 ± 1.1	4.3 ± 1.1	0.563
Creatinine, mmol/L	69.0(58.0–81.0)	67.0(56.0–80.0)	70.0(58.0-81.8)	70.0(59.0–82.0)	0.034
Glycemia, mmol/L	5.2 (4.7–6.3)	5.4 (4.7-7.0)	5.2 (4.6–6.2)	5.1 (4.6–6.1)	< 0.001
*Stent information*					
Length of stent, mm	50.1 ± 32.6	53.7 ± 32.5	57.2 ± 35.4	39.8 ± 27.0	< 0.001
Left main stem, n (%)	86 (3.4%)	13 (1.6%)	22 (2.7%)	51 (5.9%)	< 0.001
LAD, n (%)	2084 (82.6%)	697 (83.3%)	717 (86.8%)	670 (78.0%)	< 0.001
LCX, n (%)	1217 (48.3%)	501 (59.9%)	452 (54.7%)	264 (30.7%)	< 0.001
RCA, n (%)	1249 (49.5%)	399 (47.7%)	444 (53.8%)	406 (47.3%)	0.012
Bifurcation lesion, n (%)	443 (17.6%)	159 (19.0%)	142 (17.2%)	142 (16.5%)	0.387
Ostial lesions, n (%)	274 (10.9%)	76 (9.1%)	92 (11.1%)	106 (12.3%)	0.093
CTO, n (%)	224 (8.9%)	94 (11.2%)	68 (8.2%)	62 (7.2%)	0.011
*Stent type*					< 0.001
SES, n (%)	1643 (65.2%)	491 (58.7%)	582 (70.5%)	570 (66.4%)	
PES, n (%)	502 (19.9%)	184 (22.0%)	122 (14.8%)	196 (22.8%)	
BMS, n (%)	377 (14.9%)	162 (19.4%)	122 (14.8%)	92 (10.8%)	
*Clinical presentation*					0.317
Urgent PCI, n (%)	99 (3.9%)	31 (3.7%)	30 (3.6%)	38 (4.4%)	
Delayed PCI, n (%)	519 (20.6%)	169 (20.2%)	179 (21.7%)	171 (19.9%)	
NSTE-ACS, n (%)	1488 (59.0%)	493 (58.9%)	468 (56.7%)	527 (61.4%)	
SA, n (%)	416 (16.5%)	144 (17.2%)	149 (18.0%)	123 (14.3%)	

TVR, target vessel revascularization; PVD, Peripheral vascular disease; ACS, acute coronary syndrome; CABG, coronary artery bypass graft; HDL-C, high-density lipoprotein cholesterol; LDL-C, low-density lipoprotein cholesterol; COPD, chronic obstructive pulmonary disease; LAD, left anterior descending artery; LCX, left circumflex artery; RCA, right coronary artery; MI, myocardial infarction; TC, total cholesterol; CTO, chronic total occlusions; SES, sirolimus-eluting stent; PES, paclitaxel-eluting stent; BMS, bare metal stent; PCI, percutaneous coronary intervention; SA, stable angina

### The association between stent diameter and TVR

The details of univariate analysis in Table 2 showed that being male, with a history of prior PCI, statin use, diameter of stent, length of stent and right coronary artery (RCA) lesion were strongly correlated with the need for TVR. As illustrated in Table 3, multivariate analysis demonstrated that stent diameter was an independent predictor of TVR. In the non-adjusted model (Model 1), stent diameter was positively associated with TVR (continuous: OR 0.485, 95% CI 0.305–0.773, P = 0.002; categorical variable: T2 vs. T1, OR 0.541, 95% CI 0.348–0.843; T3 vs. T1, OR 0.520, 95% CI 0.334–0.809; P for trend = 0.005). After adjusting for sex, age, diabetes mellitus and hypertension in Model 2, a larger stent diameter was less likely to need TVR (continuous: OR 0.469, 95% CI 0.292–0.756, P = 0.002; categorical variable: T2 vs. T1, OR 0.528, 95% CI 0.339–0.824; T3 vs. T1, OR 0.508, 95% CI 0.324–0.797; P for trend = 0.003). Furthermore, this association remained stable when sex, age, diabetes mellitus, hypertension, atrial fibrillation, stroke, smoking, prior PCI, prior coronary artery bypass grafting, chronic obstructive pulmonary disease, RCA, statin use, length of stent, stent type and glycemic values were adjusted in Model 3 (continuous: OR 0.526, 95 %CI 0.306–0.902, P = 0.020; categorical variable: T2 vs. T1, OR 0.510, 95% CI 0.310–0.839; T3 vs. T1, OR 0.585, 95% CI 0.352–0.973; P for trend = 0.042).

**Table 2:** Univariate analysis

Variables	Statistics	TVR, P value
Male	1715 (68.00%)	1.54 (1.01, 2.35) 0.0473
Age	59.97 ± 11.09	1.01 (0.99, 1.02) 0.5222
Hypertension	1240 (49.19%)	1.07 (0.74, 1.54) 0.7115
Diabetes mellitus	520 (20.63%)	1.15 (0.75, 1.78) 0.5173
Smoking	811 (32.16%)	1.07 (0.73, 1.58) 0.7253
Stroke	133 (5.27%)	1.85 (0.97, 3.53) 0.0618
COPD	22 (0.87%)	3.16 (0.92, 10.82) 0.0671
OMI	233 (9.24%)	1.29 (0.73, 2.29) 0.3830
Atrial fibrillation	50 (1.98%)	2.24 (0.87, 5.74) 0.0941
Heart failure	294 (11.68%)	1.33 (0.79, 2.23) 0.2801
Prior PCI	169 (6.70%)	2.58 (1.52, 4.36) 0.0004
Prior CABG	21 (0.83%)	0.98 (0.13, 7.39) 0.9871
LDL-C	2.67 ± 0.94	1.05 (0.86, 1.29) 0.6295
HDL-C	1.06 ± 0.32	0.81 (0.42, 1.54) 0.5180
TC	4.26 ± 1.06	1.14 (0.95, 1.35) 0.1521
Statin	2293 (90.92%)	0.56 (0.33, 0.94) 0.0273
Clopidogrel	2415 (95.87%)	1.22 (0.44, 3.38) 0.6993
Aspirin	2487 (98.69%)	0.79 (0.19, 3.32) 0.7431
Diameter of stent	3.07 ± 0.43	0.49 (0.30, 0.77) 0.0023
Length of stent	50.12 ± 32.63	1.01 (1.00, 1.01) 0.0003
Bifurcation lesion	443 (17.57%)	1.36 (0.87, 2.11) 0.1758
Ostial lesion	274 (10.86%)	0.89 (0.48, 1.64) 0.7084
Total chronic occlusions	224 (8.88%)	1.35 (0.76, 2.40) 0.3037
Occulsion	329 (13.05%)	0.72 (0.39, 1.32) 0.2828
LM	86 (3.41%)	1.50 (0.64, 3.51) 0.3500
LAD	2084 (82.63%)	1.23 (0.74, 2.04) 0.4354
LCX	1217 (48.26%)	1.28 (0.89, 1.84) 0.1864
RCA	1249 (49.52%)	1.50 (1.03, 2.17) 0.0326

TVR, target vessel revascularization; COPD, chronic obstructive pulmonary disease; OMI, old myocardial infarction; PCI, percutaneous coronary intervention; CABG, coronary artery bypass grafting; LDL-C, low-density lipoprotein cholesterol; HDL-C, high-density lipoprotein cholesterol; TC, total cholesterol; LM, left main coronary artery,LAD, left anterior descending artery; LCX, left circumflex artery; RCA, right coronary artery

**Table 3:** Association of stent diameter and the incidence of TVR

	Events/ Incidence (%)	Model 1		Model 2		Model 3	
		Odds ratio (95% CI)	P Value	Odds ratio (95% CI)	P Value	Odds ratio (95% CI)	P Value
All participants (N = 2522)							
Continuous	143/5.7	0.485 (0.305, 0.773)	0.002	0.469 (0.292, 0.756)	0.002	0.526 (0.306, 0.902)	0.020
T1 (n = 837)	61/7.3	Ref.		Ref.		Ref.	
T2 (n = 826)	40/4.8	0.541 (0.348, 0.843)	0.007	0.528 (0.339, 0.824)	0.005	0.510 (0.310, 0.839)	0.008
T3 (n = 859)	42/4.9	0.520 (0.334, 0.809)	0.004	0.508 (0.324, 0.797)	0.003	0.585 (0.352, 0.973)	0.039
Group trend		0.445 (0.254, 0.780)	0.005	0.433 (0.245, 0.766)	0.004	0.510 (0.267, 0.974)	0.042

Model 1 adjust for: none. Model 2 adjust for: male; age; diabetes mellitus; hypertension. Model 3 adjust for: male; age; diabetes mellitus; hypertension; atrial fibrillation; stroke; smoking; prior PCI; prior CABG; COPD; RCA; statin; length of stent; stent type; glycemic value. Tertiles of stent diameter: T1 (≤ 2.85 mm), T2 (2.86–3.2 mm), T3 (≥ 3.21 mm)

### Subgroup analyses

**Fig. 2:**
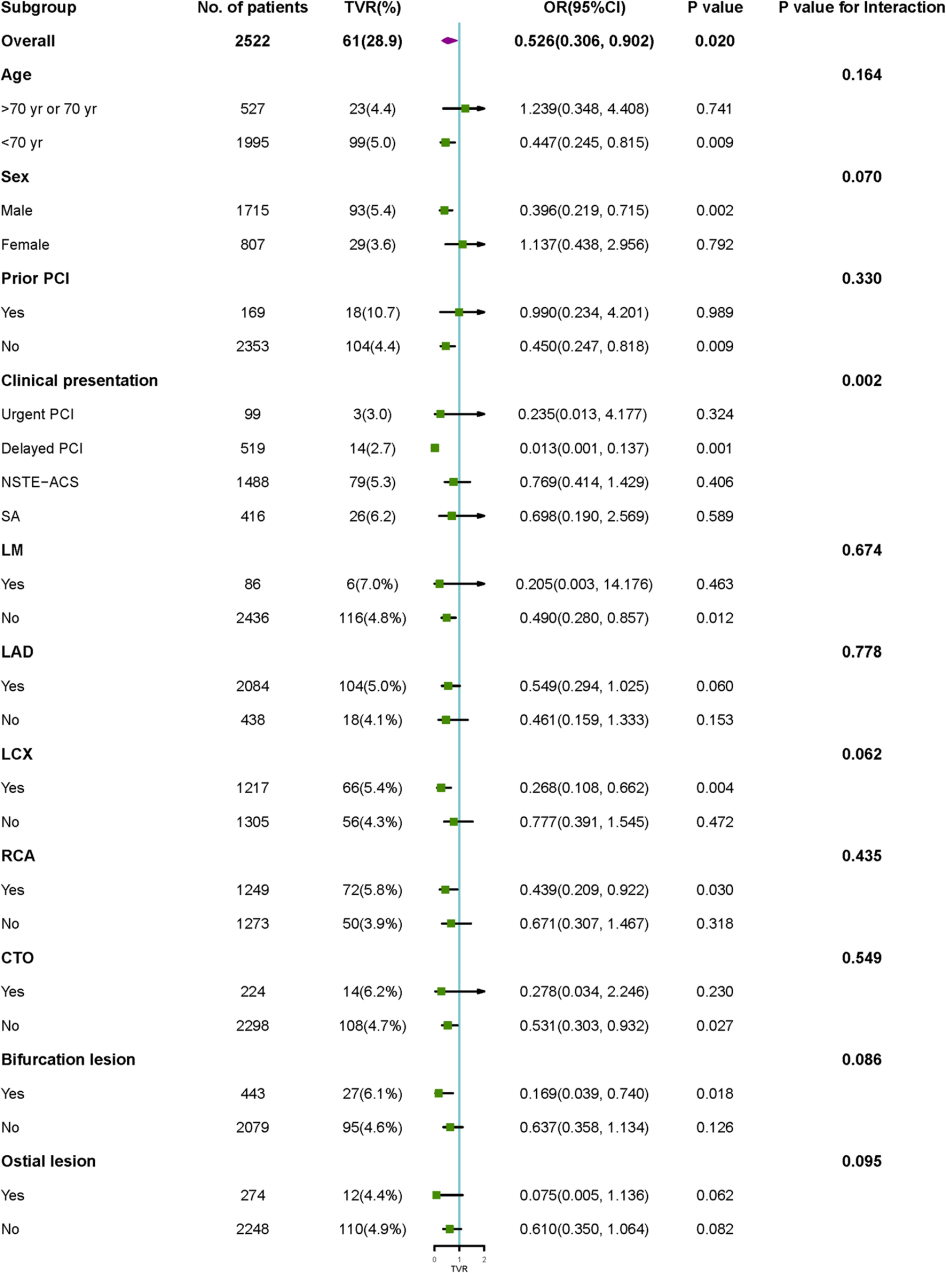
Subgroup analysis

The results of the interactions and stratified analyses are shown in Fig. 2. The results showed that an association between stent diameter and TVR was stable in different subgroups (< 70 years, male, no prior PCI, no left main coronary artery lesion, left anterior descending artery lesion, left circumflex artery lesion, right coronary artery lesion, no total chronic occlusions, bifurcation lesions and no ostial lesion), although the test for interactions was not statistically significant. Among the subgroups of clinical presentation, stent diameter was a powerful protective factor for TVR, especially in the delayed PCI group (P for interaction = 0.002).

### Piecewise linear regression model

**Fig. 3:**
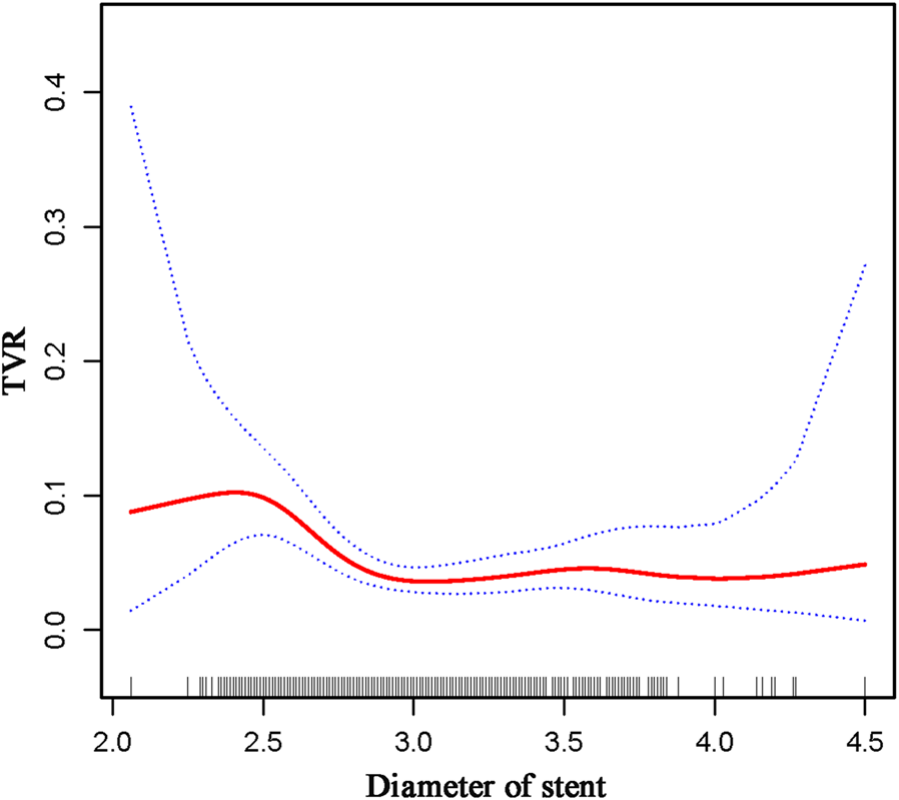
The illustrated curved line relation between stent diameter and TVR. The area between two dotted lines is expressed as a 95% CI

After adjusting for the possible factors related to TVR, a nonlinear relationship between stent diameter and TVR was observed (Table 4; Fig. 3). The occurrence of TVR decreased with an increase in stent diameter when the stent diameter ranged between 2.5 and 2.9 mm (OR 0.01, 95% CI: 0.01–0.13, P < 0.001), while the protective effect of increased stent diameter was not statistically significant in stents with diameters < 2.5 or > 2.9 mm (P = 0.285 and 0.911, respectively).

**Table 4:** Threshold effect analysis of stent diameter on TVR using piecewise liner regression

Stent diameter (mm)	OR	95% CI	P value
< 2.5	31.71	0.06 to 17982.29	0.285
2.5–2.9	0.01	0.01 to 0.13	< 0.001
> 2.9	0.95	0.40 to 2.29	0.911

Adjusted variables: male; age; diabetes mellitus; hypertension; atrial fibrillation; stroke; smoking; prior PCI; prior CABG; COPD; RCA; statin; glycemic value; length of stent and stent type

## Discussion

In this secondary analysis study, we explored the effect of stent diameter on the need for TVR. The main findings are: (1) Stent diameter was an independent predictor of TVR, after adjustment for any potential confounders. (2) The association between stent diameter and TVR remained stable in subgroups (< 70 years, male, no prior PCI, no left main coronary artery lesion, left anterior descending artery lesion, left circumflex artery lesion, right coronary artery lesion, no total chronic occlusions, bifurcation lesions and no ostial lesion). Of note, stent diameter is a powerful protective factor of TVR, especially in the delayed PCI group. (3) To be more precise, the occurrence of TVR decreased with increasing stent diameter (range: 2.5–2.9 mm); however the protective effect of increased stent diameter was not statistically significant in stent with diameter < 2.5 or > 2.9 mm.

Many studies have reported various factors that may be associated with TVR and some of them briefly mention the prognostic role of stent diameter on TVR. Hess et al. [8] analyzed the data from the CathPCI Registry and proposed that a history of prior PCI and stent length were strong TVR predictors, which was also confirmed in our study. The results of the CONSISTENT CTO study showed that diabetes may be a predictor of TVR among 210 CTO PCI patients [10]. Furthermore, hyperglycemia may affect cardiovascular events in patients with acute coronary syndrome, irrespective of whether patients have a history of diabetes [11, 12]. Hyperglycemia increases the risk of vascular damage and cardiac myocyte death through different molecular mechanisms. Therefore, glycemic values also deserve attention. Nagano et al. [13] proposed that high density lipoprotein (HDL) might be in favor of TVR after PCI in a single-center, nonrandomized study with a limited sample size. However, the effect of HDL was neither significant in our study nor in other previously reported studies with a greater number of participants. Higher HDL is known to be a protective factor for cardiovascular disease, but further studies are needed to clarify whether HDL is strongly associated with TVR. Considering that the differences in disease caused by frailty and sex are undisputed [14, 15]. Elderly men deserve more attention because of frailty and sex. Moreover, Zahn et al. [6, 16] reported that several other factors may be related to TVR, such as advanced age and prior coronary bypass.

In this study, we analyzed the relevant factors and found that stent diameter was an independent predictor of TVR, even after adjusting for these potential confounders. Furthermore, the association remained stable in all subgroups. In the delayed PCI group, the protective effect of stent diameter on TVR was particularly prominent, with a statistically significant interaction. This may be attributed to the treatment being more precise and appropriate with adequate preparation time and the use of a pre-selected program in patients with delayed PCI.

As reported in previous studies, the “bigger-is-better strategy” was popular for a long time in the bare-metal stent era [17–19]. With the development of drug-eluting stents, the subsequent restenosis reduced and the incidence of coronary perforation increased for the conception of bigger-is-better strategy, especially in chronic total occlusion, severe calcification or eccentric lesions [20, 21]. Gradually, the strategy of stent selection based on target vessel size was accepted, especially in diseases of the small vessel. Kitahara et al. [22] reported that small target vessels were more inclined to have neointimal proliferation with the implantation of an oversized stent, which may negate the benefits of larger stent. This may be the reason why the protective effect of larger stent was not found in stents with diameter < 2.5 mm in this study. In other words, the protective effect of stent size had a threshold. As shown in the piecewise linear regression model, the risk of TVR decreased with an increase in stent diameters among 2.5–2.9 mm. When the stent size was large enough (> 2.9 mm), the protective effect did not increase further and tend to be saturated.

The present study systematically explored the association between stent diameter and TVR using multivariate analysis, subgroup analyses, smooth curve fitting and piecewise linear regression models. As a result, appropriate stent diameter should be one of the most crucial considerations when clinicians choose a stent. Moreover, this study indicated that stent diameter may help identify patients at higher risk of TVR, who may require increased postoperative follow-ups. It is worth mentioning that the protective effect of larger stents still existed although it did not continue to increase significantly once the stents were large enough.

The present study had several limitations. First, the study data were obtained from a single-center database published on the Dryad Digital Repository. Although as much data as possible were included, some certain parameters (such as percent diameter stenosis and target vessel diameter), that may contribute to further exploration of risk stratification and subgroup analysis, were unavailable. Second, the accurate time intervals between PCI and TVR was not available in this retrospective study. Obviously, there are differences between TVR occur 1 year or 3 years after PCI. With the help of accurate time intervals, the association between stent diameter and TVR could be better revealed. Concerning factors associated with early restenosis and late catch-up may be different, as many factors as possible were included in multivariate analysis. Third, this association needs to be verified in other populations considering the racial heterogeneity of the coronary artery.

## Conclusions

The present study found that a larger stent diameter was a powerful protective factor of TVR in PCI patients, especially in the delayed PCI group. This “bigger-is-better” protective effect was remarkable in stents with diameter 2.5–2.9 mm, while no such association was found in stents with diameter < 2.5 mm or > 2.9 mm.

## Data Availability

The datasets generated and analysed during the current study are available in the Dryad Digital Repository, [https://datadryad.org/resource/doi:10.5061/dryad.13d31].

## References

[1] Dola J, Morawiec B, Wanha W, Nowalany-Kozielska E, Wojakowski W, Kawecki D. Results of PCI with drug-eluting stents in an all-comer population depending on vessel diameter. J Clin Med. 2020;9(2):524. 10.3390/jcm9020524 PMC7073995 32075153

[2] Zbinden R, von Felten S, Wein B, Tueller D, Kurz D, Reho I, Galatius S, Alber H, Conen D, Pfisterer M (2017). Impact of stent diameter and length on in-stent restenosis after DES vs BMS implantation in patients needing large coronary stents—a clinical and health-economic evaluation. Cardiovasc Ther.

[3] Stone G, Lansky A, Pocock S, Gersh B, Dangas G, Wong S, Witzenbichler B, Guagliumi G, Peruga J, Brodie B (2009). Paclitaxel-eluting stents versus bare-metal stents in acute myocardial infarction. N Engl J Med.

[4] Sardella G, Lucisano L, Garbo R, Pennacchi M, Cavallo E, Stio R, Calcagno S, Ugo F, Boccuzzi G, Fedele F (2016). Single-staged compared with multi-staged PCI in multivessel NSTEMI patients: the SMILE trial. J Am Coll Cardiol.

[5] Zahn R, Hamm C, Schneider S, Zeymer U, Nienaber C, Richardt G, Kelm M, Levenson B, Bonzel T, Tebbe U (2005). Incidence and predictors of target vessel revascularization and clinical event rates of the sirolimus-eluting coronary stent (results from the prospective multicenter German Cypher Stent Registry). Am J Cardiol.

[6] Zahn R, Hamm C, Schneider S, Richardt G, Kelm M, Levenson B, Bonzel T, Tebbe U, Sabin G, Nienaber C (2010). Coronary stenting with the sirolimus-eluting stent in clinical practice: final results from the prospective multicenter German Cypher Stent Registry. J Intervent Cardiol.

[7] Kastrati A, Dibra A, Mehilli J, Mayer S, Pinieck S, Pache J, Dirschinger J, Schömig A (2006). Predictive factors of restenosis after coronary implantation of sirolimus- or paclitaxel-eluting stents. Circulation.

[8] Hess C, Rao S, Dai D, Neely M, Piana R, Messenger J, Peterson E (2014). Predicting target vessel revascularization in older patients undergoing percutaneous coronary intervention in the drug-eluting stent era. Am Heart J.

[9] Yao HM, Wan YD, Zhang XJ, Shen DL, Zhang JY, Li L, Zhao LS, Sun TW (2014). Long-term follow-up results in patients undergoing percutaneous coronary intervention (PCI) with drug-eluting stents: results from a single high-volume PCI centre. BMJ Open.

[10] Walsh S, Hanratty C, McEntegart M, Strange J, Rigger J, Henriksen P, Smith E, Wilson S, Hill J, Mehmedbegovic Z (2020). Intravascular healing is not affected by approaches in contemporary CTO PCI: the CONSISTENT CTO ctudy. JACC Cardiovasc Intervent.

[11] Marfella R, Sasso F, Cacciapuoti F, Portoghese M, Rizzo M, Siniscalchi M, Carbonara O, Ferraraccio F, Torella M, Petrella A (2012). Tight glycemic control may increase regenerative potential of myocardium during acute infarction. J Clin Endocrinol Metab.

[12] Sasso F, Rinaldi L, Lascar N, Marrone A, Pafundi P, Adinolfi L, Marfella R (2018). Role of tight glycemic control during acute coronary syndrome on CV outcome in type 2 diabetes. J Diabetes Res.

[13] Nagano Y, Otake H, Toba T, Kuroda K, Shinkura Y, Tahara N, Tsukiyama Y, Yanaka K, Yamamoto H, Nagasawa A (2019). Impaired cholesterol-uptake capacity of HDL might promote target-lesion revascularization by inducing neoatherosclerosis after stent implantation. J Am Heart Assoc.

[14] Corrao S, Santalucia P, Argano C, Djade CD, Barone E, Tettamanti M, Pasina L, Franchi C, Kamal Eldin T, Marengoni A (2014). Gender-differences in disease distribution and outcome in hospitalized elderly: data from the REPOSI study. Eur J Intern Med.

[15] Marcucci M, Franchi C, Nobili A, Mannucci PM, Ardoino I, Investigators R. Defining aging phenotypes and related outcomes: clues to recognize frailty in hospitalized older patients. J Gerontol Ser A Biol Sci Med Sci. 2017;72(3):395–402. 10.1093/gerona/glw188 28364542

[16] Zahn R, Neumann F, Büttner H, Richardt G, Schneider S, Levenson B, Tebbe U, Sabin G, Nienaber C, Pfannebecker T (2012). Long-term follow-up after coronary stenting with the sirolimus-eluting stent in clinical practice: results from the prospective multi-center German Cypher Stent Registry. Clin Res Cardiol.

[17] Kastrati A, Schömig A, Elezi S, Schühlen H, Dirschinger J, Hadamitzky M, Wehinger A, Hausleiter J, Walter H, Neumann F (1997). Predictive factors of restenosis after coronary stent placement. J Am Coll Cardiol.

[18] Hoffmann R, Mintz G, Mehran R, Pichard A, Kent K, Satler L, Popma J, Wu H, Leon M (1998). Intravascular ultrasound predictors of angiographic restenosis in lesions treated with Palmaz–Schatz stents. J Am Coll Cardiol.

[19] Kasaoka S, Tobis J, Akiyama T, Reimers B, Di Mario C, Wong N, Colombo A (1998). Angiographic and intravascular ultrasound predictors of in-stent restenosis. J Am Coll Cardiol.

[20] Witzke C, Martin-Herrero F, Clarke S, Pomerantzev E, Palacios I (2004). The changing pattern of coronary perforation during percutaneous coronary intervention in the new device era. J Invasive Cardiol.

[21] Shimony A, Zahger D, Van Straten M, Shalev A, Gilutz H, Ilia R, Cafri C (2009). Incidence, risk factors, management and outcomes of coronary artery perforation during percutaneous coronary intervention. Am J Cardiol.

[22] Kitahara H, Okada K, Kimura T, Yock PG, Lansky AJ, Popma JJ, Yeung AC, Fitzgerald PJ, Honda Y. Impact of stent size selection on acute and long-term outcomes after drug-eluting stent implantation in de novo coronary lesions. Circ Cardiovasc Intervent. 2017;10(10). 10.1161/CIRCINTERVENTIONS.116.004795 28951394

